# Determination of Oocyte-Manipulation, Zygote-Manipulation, and Genome-Reprogramming Effects on the Transcriptomes of Bovine Blastocysts

**DOI:** 10.3389/fgene.2018.00143

**Published:** 2018-04-24

**Authors:** Byungkuk Min, Jung S. Park, Yong-Kook Kang

**Affiliations:** Development and Differentiation Research Center, Korea Research Institute of Bioscience Biotechnology, Daejeon, South Korea

**Keywords:** SCNT, nuclear transfer, bovine embryo, gene expression profiling, manipulation, RNA-seq, reprogramming, stemness gene

## Abstract

Somatic cell nuclear transfer (scNT) embryos suffer from damage caused by micro-operation (manipulation) and inefficient genome reprograming that hinder their normal development at different levels and in distinct ways. These two effects are inseparable in the nature of the scNT embryo, although methods to separately measure them are needed to improve scNT technology and evaluate incoming reprogramming tools. As an attempt to meet these demands, we made bovine sham nuclear-transfer (shNT) blastocysts, special embryos made with a standard nuclear-transfer procedure at the zygote stage, while retaining an intact genome. We compared their transcriptomes with those of other blastocysts derived by *in-vitro* fertilization (IVF) or scNT. Correlation analysis revealed a singularity of shNT blastocysts as they separately gathered from the others. Analysis of developmentally important genes revealed that, in shNTs, the stemness-associated differentially expressed genes (DEGs), including *OCT4*, were mostly underrepresented. Overrepresented epi-driver genes were largely associated with heterochromatin establishment and maintenance. By multilateral comparisons of their transcriptomes, we classified DEGs into three groups: 561 manipulation-associated DEGs (MADs) common to shNTs and scNTs, 764 donor genome-associated DEGs (DADs) specific to scNTs, and 1743 zygote manipulation-associated DEGs (zMADs) specific to shNTs. GO enrichment analysis generated various terms involving “cell-cell adhesion,” “translation,” and “transcription” for MADs and “cell differentiation” and “embryo implantation” for DADs. Because of the transcriptomic specificity of shNTs, we studied zMADs in detail. GO enrichment analysis with the 854 zMADs underrepresented in shNTs yielded terms related to protein and mitochondria homeostasis, while GO enrichment analysis of 889 shNT-high zMADs yielded terms related to endoplasmic reticulum stress and protein transport. We summarized the DEGs, which, with further investigation, may help improve our understanding of molecular events occurring in cloned embryos and our ability to control clonal reprogramming.

## Introduction

Somatic cell nuclear transfer (scNT) is a powerful technique to produce genetically modified animals that can be used as industrial bioreactors or models for biomedical research (Kang et al., 2003; Niemann and Lucas-Hahn, 2012). However, low cloning efficiency has limited some of these promising applications, and inefficient reprogramming of the somatic nucleus is considered the major cause of this inefficiency (Bourc'his et al., 2001; Eggan et al., 2001; Humpherys et al., 2001; Kang et al., 2001; Reik et al., 2001; Surani, 2001). Correct reprogramming changes a differentiated donor cell nucleus into a pluripotent embryonic nucleus in scNT embryos. In the presence of reprogramming errors, the scNT embryo fails to follow the embryonic gene expression program and has a faulty gene expression profile. The accumulation of gene expression errors hampers normal development of scNT embryos (Hill et al., 2000; Lanza et al., 2000; Amano et al., 2001; Eggan et al., 2001; Ono et al., 2001).

The scNT procedure involves manipulation to remove the cytoplasm, electrical fusion, or injection of a donor cell, and activation (Lagutina et al., 2007; Akagi et al., 2014). Each of these steps can have a negative impact on the developmental ability of the resulting scNT embryos (for review, see Niemann et al., 2008; Ogura et al., 2013; Smith et al., 2016). For example, in the procedure of enucleation, removal of >10% cytoplasm can decrease the developmental capability of cloned embryos and the number of blastomeres (Bowles et al., 2008; Hua et al., 2011; Panda et al., 2011). Differing from laboratory mice, which offer reproducible experimental systems through well-defined genetic backgrounds, it is very difficult, in cloning farm animals, to statistically determine the biological and/or technical factor(s) that is more responsible for the efficiency of cloning because of the considerable and uncontrollable individual differences in the quality of recipient oocytes, donor cells, and recipient females (Ogura et al., 2013). In addition to physical assaults, the scNT embryos have to overcome problems with genic and intergenic regions that are refractory to reprogramming and development. The incapability of oocyte remodeling machinery to quickly alter differentiated states of donor genome might be explained by epigenetic mechanisms that preserve global characteristics of cellular identity during cleavage. In reality, some repressive epigenetic marks such as DNA methylation and histone H3 lysine 9 or lysine 27 methylations (H3K9me and H3K27me) showed a very limited epigenetic reprogramming. This is why the developmental ability of cloned embryos are improved by the treatment of small chemicals that serve as HDAC (trichostatin A, sodium butyrate, scriptaid, and valproic acid) or DNMT (5-azacytidine) inhibitors (Kwon et al., 2017). Likewise, these factors can hinder correct reprogramming and normal development in scNT embryos. Thus, it is imperative to assess their influences separately in order to improve scNT technology and find tools that assist in efficient nuclear reprogramming. However, using existing embryo samples derived from standard scNT, it is impossible to discriminate one effect from the other because the two are intertwined.

A special kind of cloned embryo derived from a sham nuclear transfer (sham NT) can be taken as alternative. It can be prepared by manipulating a bovine zygote, not a mature oocyte to remove a part of its cytoplasm, while keeping its genomic material intact in the pronucleus. Early studies reported the developmental arrest of cloned embryos prepared from zygote enucleation in mice (McGrath and Solter, 1984a; Howlett et al., 1987; Wakayama et al., 2000). However, our preliminary result showed that the sham NT, most likely due to the possession of intact genome, maintained normal development in bovine. The resultant sham NT embryo may have half normal (normally fertilized) and half scNT characteristics. Exploiting this special feature of sham NT embryo, we aimed to separate the manipulation effect from the donor genome effect by a multilateral comparison of the gene expression profiles of normal, scNT, and sham NT embryos. With the inclusion of the sham NT embryo's transcriptome in the simple transcriptomic comparison between *in vitro* fertilization (IVF) and scNT embryos, we expect to obtain valuable information on variable sets of differentially expressed genes (DEGs) involving those in association with oocyte manipulation, zygote manipulation, or the donor genome. These lists of DEGs will serve as valuable resources to help advance our understanding of reprogramming in scNT embryos and our ability to control genome reprogramming for more efficient cloning.

## Materials and methods

### Generation of IVF blastocysts

This study was carried out in strict accordance with the recommendations in the Guide for the Care and Use of Laboratory Animals of the National Livestock Research Institute of Korea. The protocol was approved by the Committee on the Ethics of Animal Experiments of the Korea Research Institute of Bioscience and Biotechnology.

Bovine oocytes were collected from ovaries supplied by a local slaughterhouse and matured in the paraffin oil covered in vitro maturation medium for 20 h at 38.5°C with 5% CO2. The medium for oocytes maturation was prepared by combining TCM-199 (Invitrogen) supplemented with 10% (v/v) fetal bovine serum (FBS; Invitrogen), 10 μg/ml FSH-P (Folltropin-V, Vetrepharm), 0.6 mM cysteine, 0.2 mM sodium pyruvate, and 1 μg/ml estradiol-17β together. To generate IVF embryos, the matured oocytes were fertilized by incubating with 2 × 106 sperms/ml in fertilization medium at 38.5°C in 5% CO2 for 20–22 h (Park et al., 2007). After the insemination, cumulus cells were removed by gentle pipetting, and the fertilized eggs were further cultured in CR1aa supplemented with 3 mg/ml BSA (fatty acid free). After 3 days, cleaved embryos were cultured in CR1aa (with 10% FBS) for 4 days at 38.5°C in 5% CO2 (Koo et al., 2002).

### Production of somatic cell nuclear transfer and sham nuclear transfer embryos

For the generation of bovine scNT blastocysts, bovine mature oocytes were manipulated as described elsewhere (Koo et al., 2002). Oocyte manipulations including enucleation were performed by using a micromanipulator equipped with an inverted microscope (Leitz, Ernst Leitz Wetzlar GmbH). The medium containing TL-Hepes with 7.5 μg/ml cytochalasin B was used for manipulation. The first polar body and a part of the cytoplasm were removed together by a micropipette, and single cells were individually transferred to the perivitelline space of the recipient oocytes. The donor cell containing oocytes were equilibrated for 10–20 s in 50 μl of cell fusion medium and transferred into a fusion medium containing 0.01% BSA, 0.1 mM CaCl2, 0.3 M mannitol, 0.5 mM Hepes, and 0.1 mM MgCl2. The donor cells were fused into the oocytes by a single pulse of direct current of 1.6 kV/cm for 20 μs each by an Electro Cell Manipulator 2001 (BTX). After 1 h, the oocytes with no visible somatic donor cells in the perivitelline space were selected, and they were activated with 5 μM Ionomycin for 5 min, followed by treatment with 2.5 mM 6-dimethyl-aminopurine (DMAP, Sigma) in CR1aa supplemented with 10% FBS for 3.5 h at 38.5°C in 5% CO2 in air. The activated reconstructed oocytes were cultured in the same conditions as IVF embryos for 7 days until they formed blastocysts. As for donor cells, ear skin fibroblasts were obtained from an adult female or male cow and passaged 3 times in the standard culture condition as described before (Koo et al., 2002).

For generation of sham NT blastocysts, the zygotes presenting both parental pronuclei were manipulated at ~15 post-IVF h. For the precise imitation of the physical damages by enucleation, zona pellucida was partially ripped, and the polar body and a part of the underlying ooplasm were carefully removed by aspiration using a micropipette without disturbing pronuclei. The oocytes were activated, after 2 h of incubation, using 5 μM ionomycin (Sigma) for 5 min, followed by treatment of 2.5 mM DMAP in CR1aa culture media supplemented with 0.3% BSA for 3.5 h. Blastocysts were generated 7 days post-IVF or -NT. The quality of each blastocyst was assessed by Hoechst staining, and only high-quality ones at mid blastocyst stage possessing 60–80 blastomeres were chosen for transcriptomic analysis.

For chemical activation of mouse zygotes which were used to examine the effect of chemical-mediated secondary activation on their ability of normal development, fertilized mouse oocytes were collected from superovulated C57BL/6 females as described previously (Hogan, 1994). Briefly, female C57BL/6 mice at 5 weeks of age were injected with 5 IU of pregnant mare serum gonadotrophin, followed by 5 IU of human chorionic gonadotropins 48 h apart, and mated with male mice. Successful mating was determined the following morning by detection of a vaginal plug. Mouse zygotes were collected from mouse oviduct and transferred to M2 medium (Sigma) containing 0.1% (w/v) hyaluronidase to remove cumulus cells and cultured in M16 medium (Sigma) at 37°C, 5% CO_2_ in air (Yeo et al., 2005). The mouse zygotes were subjected to the same protocol of chemical activation as the bovine zygotes (see above) and cultured to the 2-cell stage. The cleaved 2-cell embryos were transferred to a pseudo-pregnant surrogate mother. Fetal development was observed at 13.5 days post coitem (dpc).

### Pico-profiling of mRNA obtained from a single blastocyst

Each single blastocyst generated by IVF, scNT, or sham NT was lysed, and mRNAs were directly isolated from the lysates using Dynabeads mRNA DIRECT Kit (Invitrogen) according to the manufacturer's recommendation. The ultra-pure mRNAs were reverse transcribed using a custom pico-profiling adapter as previously described (Min et al., 2015). First-strand cDNAs were synthesized by incubating mRNAs with 100 pmol of pico-profiling adapters and 1 μl SuperScript III enzyme (Invitrogen) in 15 μl reaction volume using a following sequential program: 18°C for 10 min, 25°C for 10 min, 37°C for 30 min, 42°C for 10 min, and 70°C for 20 min. Then, 1 μl T4 DNA polymerase (NEB) were added and further incubated at 37°C for 1 h to tag both ends of cDNAs. For amplification of the cDNAs, 15–20 cycles of PCR was performed 94°C for 2 min, 70°C for 5 min using an anchor primer. The amplified cDNA libraries were digested by MlyI enzyme (NEB) overnight and then purified using Agencourt AMPure XP beads (Beckman Coulter) before used for NGS library construction.

### RNA-seq library generation

A total of 35 RNA-seq libraries (12 IVF, 12 scNT, and 11 shNT blastocysts) were constructed using TruSeq DNA Sample Preparation kit (Illumina) (Min et al., 2015, 2017). Two hundred nano grams of pico-profiled amplicons were applied to the End-repair reactions by incubating with End Repair Mix (Illumina) at 30°C for 2 h. The reactions were purified with AMPure XP beads (Beckman). Then, A-Tailing Mix (Illumina) was added to the samples, and the mixtures were incubated at 37°C for 2 h. Next, indexed adapter was added into each sample with Ligation Mix (Illumina) and the mixtures were incubated overnight at 16°C. Ligates were purified using AMPure XP bead (Bechman). For size selection, adapter ligated DNA samples were loaded onto Pippin Prep (Sage Science), and DNA fragments between 300 and 500 bp were isolated. Finally, the isolated DNAs were enriched by 15–18 cycles of PCR reaction. The enriched DNA fragments were purified and sequenced (2 × 100) using HiSeq2500. All raw sequencing data are available at Gene Expression Omnibus (GSE95311) and our previous publication (PMID: 28443134).

### Estimation of gene expression levels and bioinformatic analyses

Raw read sequences (2 × 100 bp) from the HiSeq2000 system were preprocessed to remove NGS artifacts such as adapter sequences and low quality bases by “trimgalore,” and the trimmed reads were aligned on the reference genome (UMD3.1) using TopHat (Kim et al., 2013) with the seed length (L) of 10 to increase specificity. We followed the cufflinks pipeline (Trapnell et al., 2012) to estimate the gene expression levels and identify the DEGs. All cufflinks suits were operated with their default options unless noted otherwise. First, transcriptomes of individual samples were assembled using “cufflinks” with –G option to obtain the expression levels of only known genes due to the incomplete genome assembly of bovine. The transcriptomes were merged into one by “cuffmerge,” and differential expression (DE) analysis was performed by “cuffdiff.” Pearson correlation coefficients between samples and groups were calculated by “cor,” an R function. Gene ontology and KEGG pathway analysis was performed using DAVID Bioinformatics Resources 6.8 (Huang da et al., 2009). Weighted gene co-expression network analysis (WGCNA) was executed as described elsewhere using the power of β = 5 (Langfelder and Horvath, 2008; Xue et al., 2013). All plots in this study were produced by R scripts, MS EXCEL, and Origin 8.

## Results

### Production of shNT blastocysts with distinctive transcriptomes

**Figure 1:**
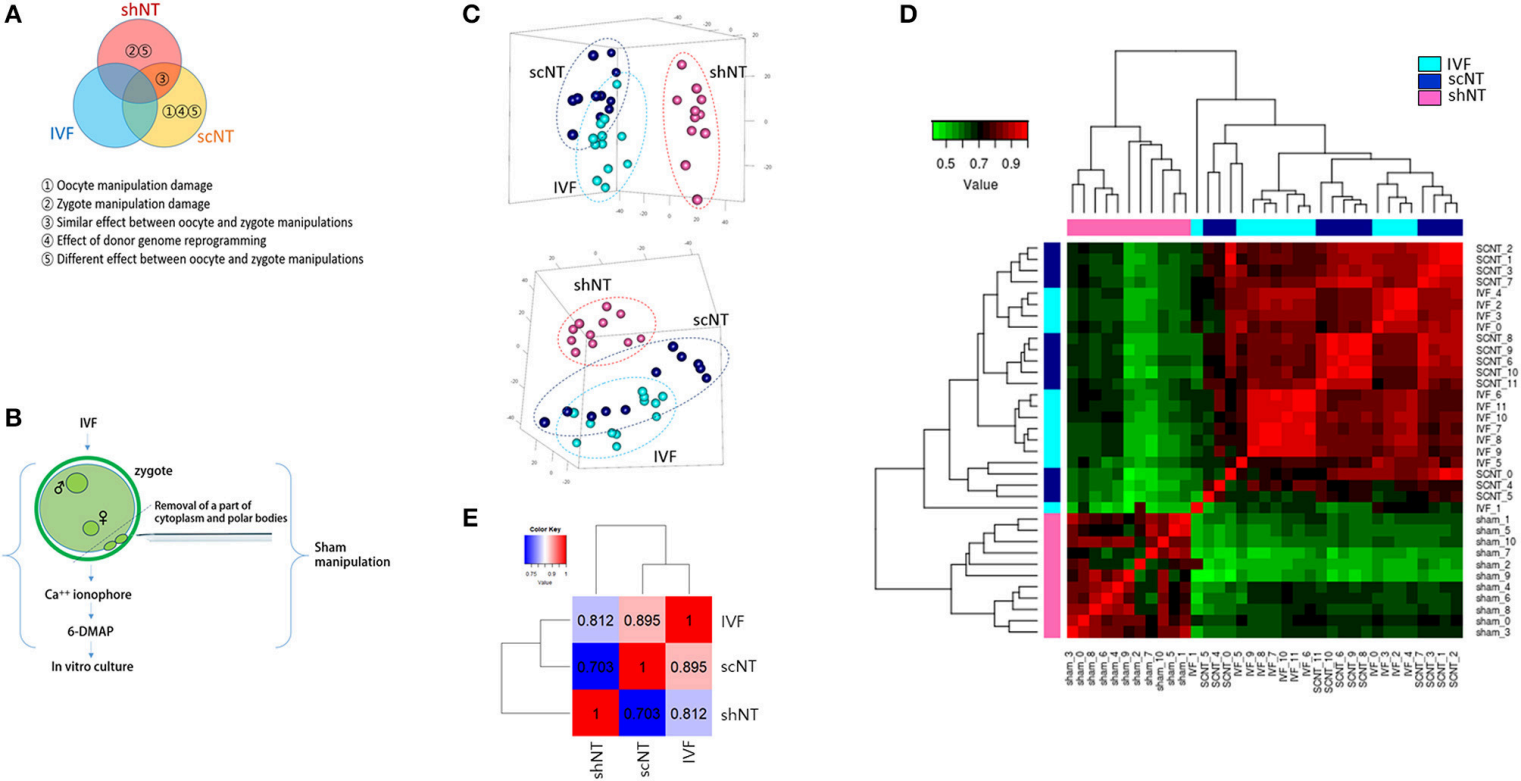
Correlation analysis of the transcriptomes obtained from bovine IVF, scNT, and shNT blastocysts. **(A)** Classification of differentially expressed genes (DEGs) to be identified from transcriptomic comparisons of IVF, scNT, and shNT blastocyst groups. Definitions for the classified DEGs are listed below. **(B)** Production of shNT blastocysts. **(C)** Principal component analysis (PCA) showing a distant clustering of shNT blastocysts (dark pink) from other IVF (cyan) and scNT blastocysts (dark blue). Each blastocyst group is circled in different colors. **(D)** Heat-map of Pearson correlations between individual blastocysts. The color key represents the range of transcriptomic correlation coefficients (r) between individual blastocysts. **(E)** Pearson correlation between blastocyst groups. Correlation coefficient values between the blastocyst groups (r) are indicated.

We compared the transcriptomes of three different kinds of bovine blastocysts derived by IVF, scNT, and shNT procedures. The comparisons enabled us to separately examine the effects of various NT-involved factors such as the manipulation, partial removal of oocyte/zygote cytoplasm, and donor genome reprogramming (Figure 1A). We analyzed previous Pip-seq data (Min et al., 2017) obtained from individual blastocysts after PCR-mediated amplification of embryonic transcripts by pico-profiling (Min et al., 2015, 2016). Each blastocyst group comprised 12 blastocysts, with six males and six females (with the exception of five female shNTs). We examined the gene expression profiles, particularly of shNT blastocysts, to determine how the transcriptome differed from IVFs and scNTs. The shNTs were prepared as depicted in Figure 1B. Briefly, the zygote with small pronuclei (~15 h after sperm exposure) was put through the NT procedure, in which a part of its cytoplasm was removed carefully to avoid losing genomic material. Therefore, the zygote and its descendants kept the genomic material intact in the nucleus. Meanwhile, the sham-manipulated zygotes were treated with the chemicals for artificial activation (DMAP and Ionomycin), which aimed to reiterate NT procedure equally in the sham zygotes. The *in vitro* developmental rates of the IVF, scNT, and shNT groups are summarized in the Figure S1A. Only high-quality blastocysts at mid blastocyst stage possessing 60–80 blastomeres were chosen for transcriptomic analysis (Figure S1B). Meanwhile, we examined whether the repeated activation (fertilization and artificial activation) could be intolerable and would be catastrophic to the bovine zygotes as it is thought. When we equally treated mouse zygotes with the same activating chemicals, we observed that mouse zygotes could normally develop as the control embryos, indicating that the repeated activation is not disastrous to embryo development (Figure S1C). Manipulating the zygotes instead of the oocytes and retaining an intact zygote genome were the only differences in the sham NT protocol compared with the standard NT protocol (Park et al., 2007; Kwon et al., 2015).

The number of genes expressed in IVFs, scNTs, and shNTs was 14,095, 13,840, and 13,471, respectively. Although slightly lower in the shNTs, the number of genes was not significantly different (*p* = 0.067; one-way ANOVA) among the three groups. From principal component analysis (PCA), we found that the shNTs separately grouped from the IVFs and the scNTs, while the latter two associated together (Figure 1C). Assessment of correlations by unsupervised clustering revealed that shNTs were weakly correlated with IVFs and scNTs (Figure 1D). Pearson correlation between the groups showed that the highest correlation came from the IVF-scNT match (*r*^2^ = 0.895) and the next highest from IVF-shNT (0.812) and shNT-scNT matches (0.703; Figure 1E). These data indicate that the transcriptomes of shNTs are very distinct from those of IVFs and scNTs.

### Identification of the manipulation-associated DEGs (MADs) and the donor genome-associated DEGs (DADs)

We identified 1,873 DEGs (fold-change > 2.0 and *p*-value < 0.05) between shNTs and IVFs (Figure S2). These were assumed to reflect the physical (from zygote manipulation) and biochemical (from a partial loss of the cytoplasm) damage in the shNTs (see Figure 1A). Among them, a half (937 DEGs) were underrepresented in shNTs, whereas the other half (936 DEGs) were overrepresented. This was greater than the number of DEGs obtained from the IVF-scNT comparison (256 scNT-low and 183 scNT-high DEGs) and from the shNT-scNT comparison (889 shNT-high and 819 shNT-low DEGs). These results suggest that the IVFs-shNTs comparison has the largest transcriptomic difference. The DEG information is summarized in Supplementary File 1.

The DEGs detected between IVFs and shNTs could be either manipulation-dependent or -independent. This could be discriminated by including an additional set of DEGs, the IVF-scNT DEGs, in the simultaneous comparison. Given that shNTs have the same nuclear material as IVFs, those DEGs that were commonly detected in the IVF-scNT and IVF-shNT DEG sets are likely to be manipulation-dependent. Here, we call these “manipulation-associated DEGs (MADs).” In contrast, those DEGs present in the IVF-scNT DEG set but absent from the IVF-shNT set may be donor genome-dependent. These present as a signature of inefficient reprogramming, which we call the “donor genome-associated DEGs (DADs).”

**Figure 2:**
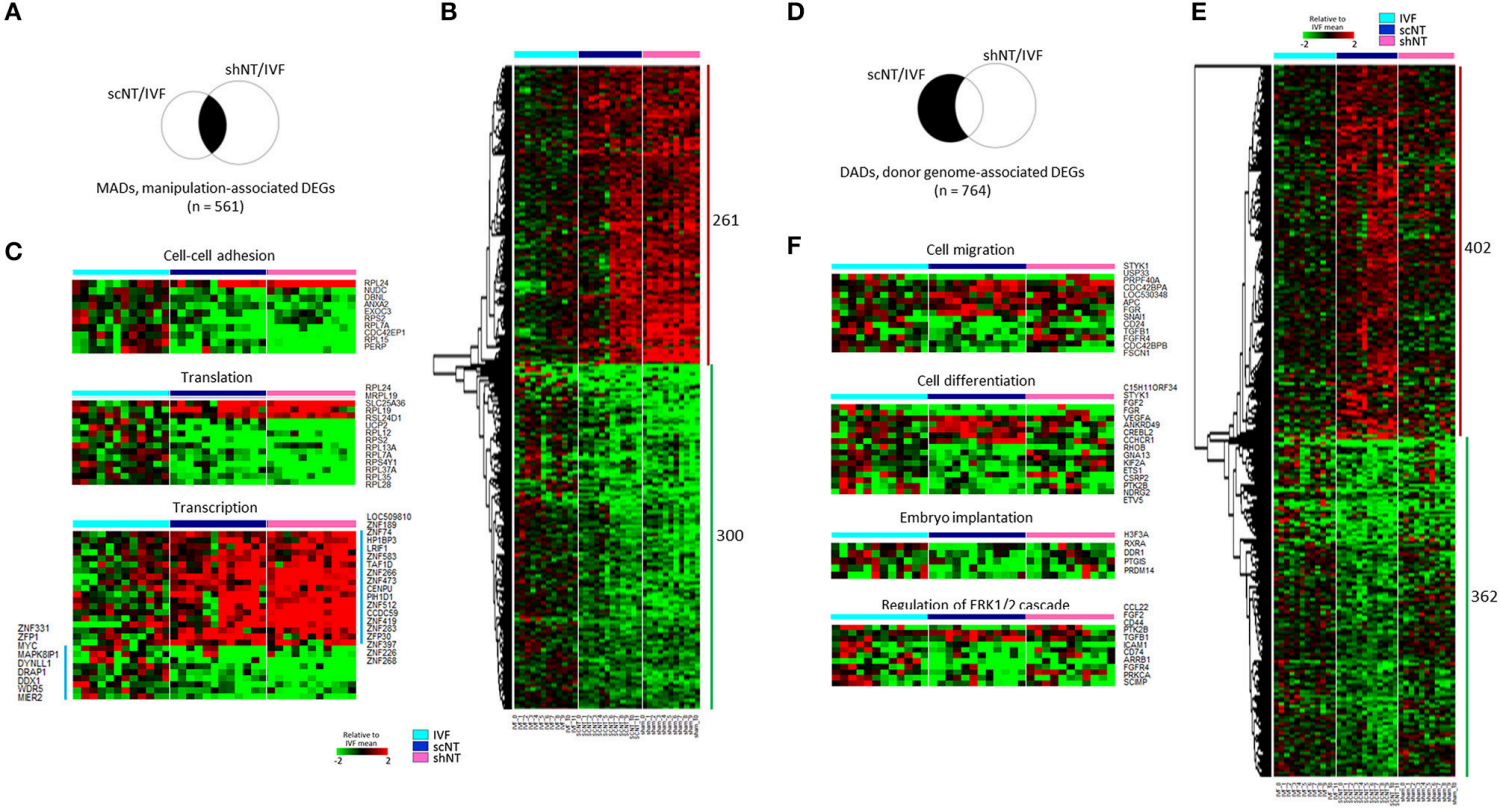
Classification of DEGs to manipulation-associated (MADs) and donor genome-associated DEGs (DADs) via multilateral comparison of transcriptomes of IVF, scNT, and shNT blastocyst groups. **(A)** Illustrative definition of MADs, a set of DEGs (*n* = 561) that are commonly detected in the IVF-scNT DEG set and the IVF-shNT DEG set as denoted by shaded areas on the Venn diagram. **(B)** Heat-map of relative expression levels against the IVF mean of 261 IVF-high and 300 IVF-low MADs in IVF (cyan), scNT (blue), and shNT (pink) blastocyst samples. **(C)** Representative result of gene ontology (GO, “biological process” category) analysis with the MADs. **(D)** Illustrative definition of DADs, a set of DEGs (*n* = 764) that are specific to scNTs in the IVF-scNT DEG set and the IVF-shNT DEG set, as denoted by shaded areas on the Venn diagram. **(E)** Heat-map of relative expression levels against the IVF mean of 402 scNT-high and 362 scNT-low DADs. **(F)** Representative result of GO enrichment analysis with DADs.

As shown in the Venn diagram in Figure 2A, 561 DEGs (*p* < 0.05) were identified as MADs. Heat-map analysis shows a collection of 261 overrepresented and 300 underrepresented MADs, in which the levels differed from those of the IVFs (Figure 2B). GO enrichment analysis yielded several terms in the “biological process” category, which we summarize in Table 1. Some representative terms such as “cell-cell adhesion” (*p* = 1.98 × 10^−4^), “translation” (*p* = 2.55 × 10^−3^), and “transcription” (*p* = 0.031) are shown as expression heat-maps with gene symbols in Figure 2C. MADs representing “cell-cell adhesion” and “translation” functions were generally underrepresented, whereas those representing “transcription” functions were largely overrepresented in the scNTs and shNTs.

**Table 1:** Gene ontology analysis using manipulation-associated and donor genome-associated DEGs.

	**Count**	***p*-value**	**Genes**
**MANIPULATION-ASSOCIATED DEGS (MADS)**			
Cell-cell adhesion	10	1.98E-04	*DBNL, CDC42EP1, RPL15, EXOC3, RPL24, RPL7A, RPS2, PERP, NUDC, ANXA2*
Translation	14	2.55E-03	*RPL19, RPL35, RPL24, RPS2, RPL28, UCP2, RPL13A, SLC25A36, MRPL19, RPS4Y1, RPL37A, RPL12, RSL24D1, RPL7A*
Assembly of large subunit precursor of preribosome	3	2.77E-03	*EIF6, RPL24, RSL24D1*
RNA secondary structure unwinding	6	7.28E-03	*DDX17, EIF4A2, DDX1, DDX54, DDX4, DDX42*
Fatty acid biosynthetic process	5	1.44E-02	*SC5D, SCD, FA2H, CBR4, DEGS1*
Positive regulation of DNA-templated transcription, initiation	3	1.79E-02	*BCLAF1, JUN, FOSL1*
Ribosomal large subunit assembly	4	2.23E-02	*RPL24, RPL12, RSL24D1, MRPL20*
Regulation of transcription, DNA-templated	28	3.14E-02	*ZNF583, HP1BP3, TAF1D, CCDC59, ZNF331, ZNF512, LOC509810, ZNF226, MIER2, DYNLL1, DRAP1, ZNF397, ZNF473, ZNF74, ZNF268, MYC, ZNF266, ZNF283, LRIF1, ZFP30, WDR5, DDX1, ZFP1, ZNF189, PIH1D1, ZNF419, CENPU, MAPK8IP1*
Negative regulation of translation	5	3.37E-02	*RPL13A, DAPK3, GAPDH, GIGYF2, TOB1*
glycolytic process	4	4.02E-02	*ADPGK, HKDC1, GAPDH, ENO1*
Negative regulation of neurogenesis	3	4.32E-02	*BRINP1, DYNLT1, HOOK3*
**DONOR GENOME-ASSOCIATED DEGS (DADS)**			
Positive regulation of endothelial cell migration	7	7.87E-04	*PRKCA, PTK2B, ETS1, GATA3, VEGFA, RHOB, FGF2*
Response to virus	8	1.22E-03	*HYAL1, ZC3HAV1, GATA3, HSPB1, EEF1G, CDK6, MX1, DHX58*
Positive regulation of Rho protein signal transduction	5	1.51E-03	*APOA1, ARRB1, GPR18, ROBO1, LPAR1*
Cell migration	13	1.72E-03	*FGFR4, FGR, FSCN1, LOC530348, SNAI1, TGFB1, STYK1, CDC42BPA, CD24, USP33, PRPF40A, CDC42BPB, APC*
Positive regulation of angiogenesis	10	4.81E-03	*PRKCA, HYAL1, PTGIS, PTK2B, VEGFA, RHOB, HSPB1, ERAP1, CHRNA7, FGF2*
Neuron projection development	9	5.75E-03	*APP, PTPRM, BTG2, NEDD4, BLOC1S3, CD24, HERC1, SRF*
Negative regulation of cell cycle	5	6.09E-03	*GATA3, NR4A1, RHOB, CDK6, TGFB1*
Ventricular septum development	5	6.09E-03	*LTBP1, SALL4, GATA3, HEG1, NPRL3*
Cell adhesion	15	6.17E-03	*CTNNAL1, PRKCA, RPSA, ICAM1, ATP1B2, NOV, ALCAM, IGSF11, CD44, PTK2B, TGFBI, COL12A1, RHOB, DPP4, SPON1*
Protein dephosphorylation	6	7.57E-03	*PTPRM, CPPED1, PPM1K, PPP2CB, PPP3R1, PPTC7*
regulation of cytokinesis	5	8.67E-03	*PLK3, PIK3C3, BRCA2, KLHL21, PRPF40A*
Positive regulation of mitotic cell cycle	5	1.37E-02	*PRKCA, APP, USP2, PKN2, BRCA2*
Embryo implantation	5	1.37E-02	*DDR1, PRDM14, PTGIS, RXRA, H3F3A*
Negative regulation of inflammatory response	7	1.74E-02	*NOV, APOA1, PTGIS, ETS1, GATA3, FEM1A, ADA*
Regulation of cell shape	10	1.81E-02	*GNA13, ANXA7, EPB41L3, FGR, TTBK2, PTK2B, VEGFA, LPAR1, SLC9A3R1, PRPF40A*
Positive regulation of ERK1 and ERK2 cascade	11	2.60E-02	*PRKCA, ICAM1, CCL22, FGFR4, CD44, ARRB1, PTK2B, FGF2, SCIMP, CD74, TGFB1*
Cell differentiation	16	2.73E-02	*GNA13, FGR, CSRP2, CCHCR1, STYK1, ANKRD49, ETS1, PTK2B, VEGFA, RHOB, NDRG2, C15H11ORF34, ETV5, FGF2, CREBL2, KIF2A*
Positive regulation of fibroblast proliferation	5	4.62E-02	*CDK6, LIG4, ITGB3, CD74, TGFB1*

The DADs outnumbered the MADs (Figure 2D). Seven hundred and sixty-four DEGs were identified as DADs, with 402 scNT-high and 362 scNT-low DADs (Figure 2E). GO enrichment analysis yielded various “biological process” terms, which are summarized in Table 1. Most (11/16) of the DADs belonging to “cell differentiation” functions were underrepresented in scNTs compared to that in IVFs and shNTs (*p* = 0.027; Figure 2F). DADs associated with “embryo implantation” functions also showed decreased expression in scNTs (*p* = 0.014). Further information on the MADs and DADs is presented in Supplementary File 2. Additionally, we examined the enrichment of KEGG pathways associated with MADs or DADs and found enrichment of pathways involved in protein synthesis such as “ribosome” and “ribosome biogenesis in eukaryotes” for MADs, while DADs showed significant association with various disease related pathways including Huntington's disease and Alzheimer's disease. The full list of significantly enriched pathways are presented in Supplementary File 2.

### Identification of zygote manipulation-associated DEGs (ZMADs) and their implications in development

**Figure 3:**
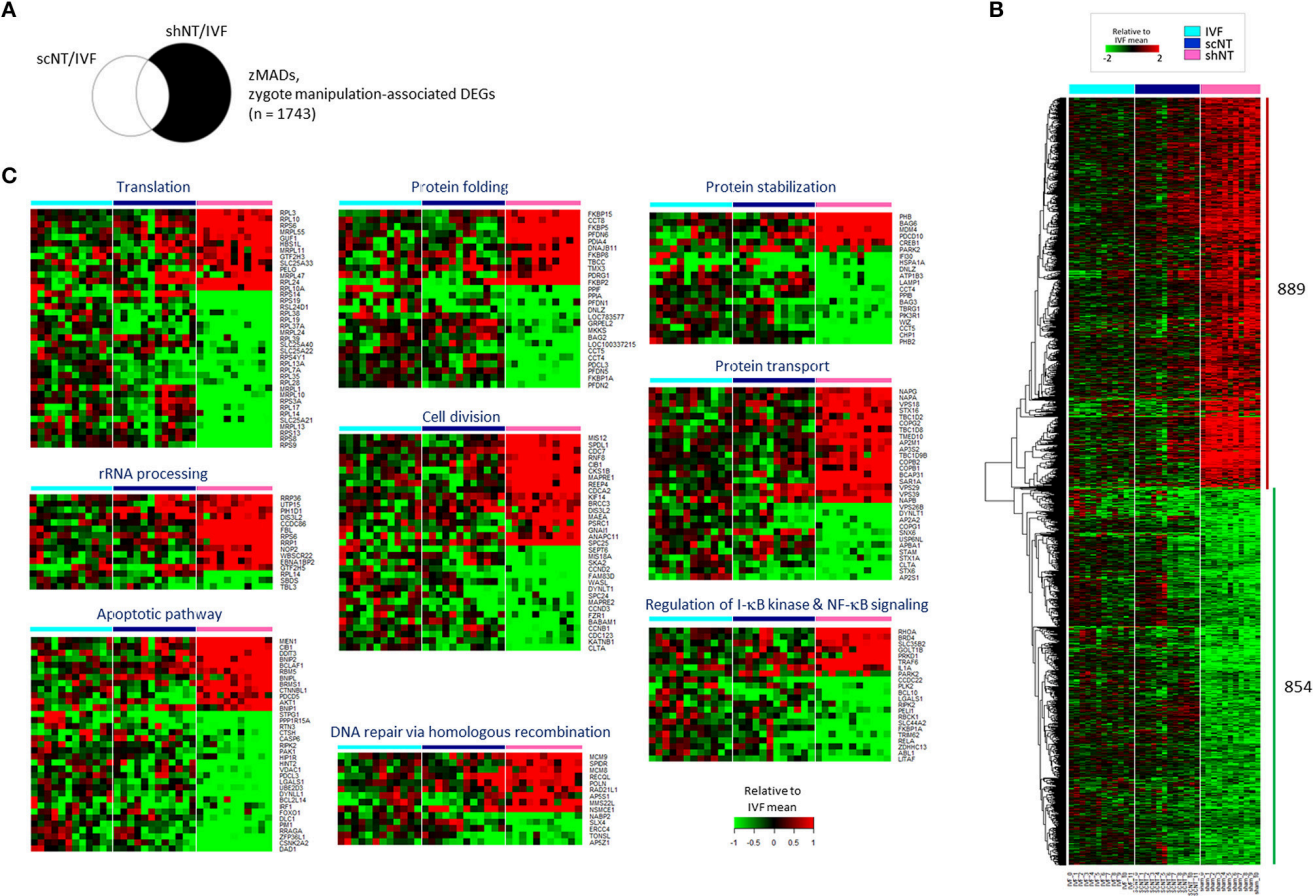
Identification of zygote manipulation-associated DEGs (zMADs) from the comparison of transcriptomes of IVF, scNT, and shNT blastocyst groups. **(A)** Illustrative definition of zMADs, a set of DEGs (*n* = 1743) that are specific to shNTs in the IVF-scNT DEG set and the IVF-shNT DEG set, as denoted by shaded areas on the Venn diagram. **(B)** Heat-map of relative expression levels against the IVF mean of 889 shNT-high and 854 shNT-low zMADs in IVF (cyan), scNT (blue), and shNT (pink) blastocyst samples. **(C)** Representative result of GO enrichment analysis with zMADs.

A relatively large number of genes (*n* = 2,921, *p* < 0.05) were found as shNT-specific DEGs (Figure 3A), which was too large in number to run a GO engine. The output of GO analysis with the 2,921 genes that account for nearly one-third of expressed genes and are presumably implicated in that much of biological processes would be unambiguously enormous and confusing, thus definitely irrelevant to find significant and valuable terms from the result (see Supplementary File 2 for GO result using 2,921 genes). Therefore, we raised the stringency of DEG selection (*p* < 0.05 plus a fold-change > 2.0). Under these new criteria, 889 shNT-high and 854 shNT-low DEGs were identified (Figure 3B). They are listed in Supplementary File 2. These shNT-specific DEGs reflect the effect of zygote manipulation (Figure 1A) and therefore we call them “zygote manipulation-associated DEGs (zMADs).” GO enrichment analysis yielded a variety of terms, some of which are shown in Figure 3C. Categories most highly ranked were those implicated in protein processing such as “translation” (*p* = 1.40 × 10^−6^), “protein folding” (*p* = 1.58 × 10^−5^), and “protein stabilization” (*p* = 2.98 × 10^−3^). DEGs implicated in “rRNA processing” (*p* = 7.24 × 10^−5^) and “double-strand break repair” (*p* = 2.12 × 10^−3^) functions were characterized by higher expression levels in the shNTs. “Apoptosis pathway” (*n* = 35; *p* = 7.57 × 10^−4^) and “I-κB kinase- and NF-κB signaling” (*n* = 22, *p* = 7.84 × 10^−3^) functions were also detected with statistical significance.

**Figure 4:**
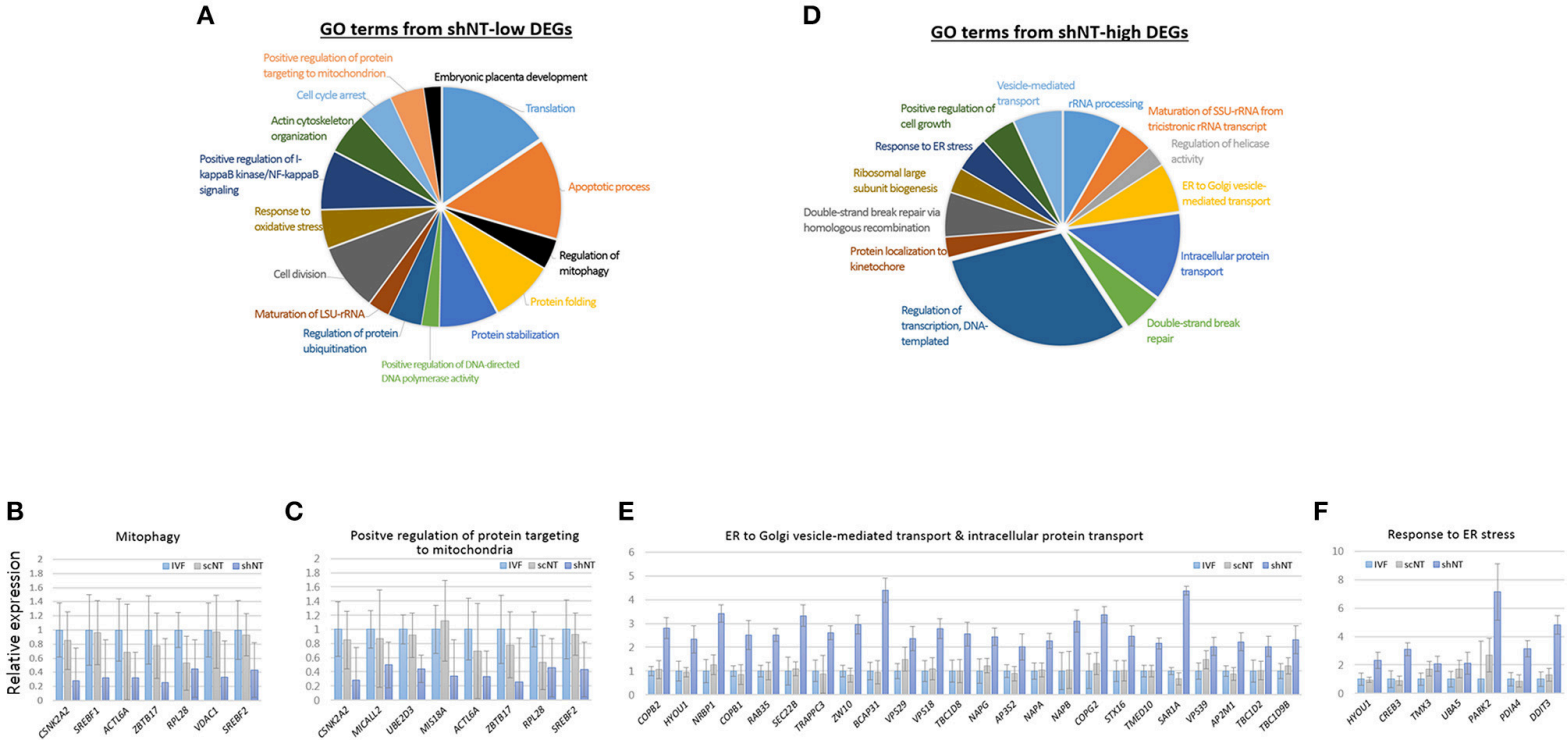
Characteristics of zygote manipulation-associated DEGs (zMADs). **(A,D)** Pie charts showing GO (“biological processes”) terms enriched in shNT-low **(A)** and shNT-high **(D)** zMADs. **(B,C)** and **(E,F)**, Relative expression levels of genes that belong to the indicated GO terms. Error bars, standard deviation. Statistics, Student's *t*-test.

Because of the opposite expression patterns among genes in the GO terms (Figure 3C), we re-generated GO terms separately using the shNT-high and the shNT-low zMADs (Table 2). Among the GO terms generated from the shNT-low zMADs, several were associated with protein synthesis and protein stability, such as the “translation,” “protein folding,” “protein stabilization,” and “protein ubiquitination” terms (Figure 4A). Downregulation of genes associated with these terms may disturb protein homeostasis and related cellular processes, suggesting an innate weakness of the shNTs in terms of protein manufacturing and processing. Other GO terms frequently shown from the analysis of shNT-low zMADs were related to mitochondrial functions. These included the “regulation of mitophagy,” “mitochondrial translational initiation/elongation,” “positive regulation of protein targeting to mitochondria,” and “protein import into mitochondrial matrix” (refer to Table 2 for those not shown in Figure 4A). Early embryos rely on the mitochondrial pool inherited from the oocyte and it is not until the blastocyst stage that mitochondria commence replication (Dumollard et al., 2007). Therefore, the reduced pool of mitochondria left in the recipient cytoplasm can interfere with proper development of the shNT embryo. Genes in the term “regulation of mitophagy,” a process of autophagy-mediated selective degradation of defective mitochondria (Lemasters, 2005), were expressed in shNTs at only 34% of the level in IVFs (Figure 4B). From this, we infer that shNTs may keep saving normal-looking, but functionally defective, mitochondria in the cytoplasm. A similar level of downregulation was observed with genes in the “protein targeting to mitochondria” term (Figure 4C). Given that the same sets of genes are expressed in scNTs at ~ 85% of the level of expression in IVFs, we assume that the zygote manipulation is more detrimental to mitochondrial homeostasis in early development.

**Table 2:** Gene ontology analysis using zygote manipulation-associated DEGs.

**GO Term**	**Count**	***p*-value**	**Genes**
rRNA processing	12	9.48E-06	*EBNA1BP2, CCDC86, PIH1D1, RRP1, NOP2, RRP36, UTP15, DIS3L2, WBSCR22, GTF2H5, RPS6, FBL*
Maturation of SSU-rRNA from tricistronic rRNA transcript	7	1.74E-03	*UTP23, UTP3, DCAF13, TSR1, UTP6, CCDC59, WDR46*
Regulation of helicase activity	4	1.93E-03	*MSH6, MSH3, MSH2, POT1*
ER to Golgi vesicle-mediated transport	10	2.81E-03	*COPB2, HYOU1, IER3IP1, NRBP1, COPB1, RAB35, SEC22B, TRAPPC3, ZW10, BCAP31*
Intracellular protein transport	18	3.91E-03	*VPS29, VPS18, TBC1D8, NAPG, AP3S2, NAPA, NAPB, BCAP31, COPB2, COPG2, COPB1, STX16, TMED10, SAR1A, VPS39, AP2M1, TBC1D2, TBC1D9B*
Double-strand break repair	8	4.42E-03	*RNF8, RAD21L1, BRCC3, APLF, MSH2, TDP2, WRN, SOD1*
Regulation of transcription, DNA-templated	44	4.91E-03	*ZNF583, TAF1D, ATP1B4, CNOT3, WBSCR22, CCDC59, LOC509810, ZNF512, LOC787057, PRMT7, ZNF300, PRMT5, ZNF397, CSDE1, ZNF473, ZNF74, MKX, ASF1B, ZNF268, ZNF266, CIITA, ZNF398, TFIP11, ZNF283, L3MBTL2, LOC613546, LRIF1, ZFP30, ZNF8, GTF2H3, GTF2H5, EXO5, DDIT3, CCNL2, TEFM, PIH1D1, ZNF419, SRFBP1, ZNF197, ZNF214, APBB3, HDAC8, COMMD6, VPS25*
Protein localization to kinetochore	4	5.02E-03	*SPDL1, AURKB, MIS12, ZW10*
Double-strand break repair via homologous recombination	9	5.30E-03	*MCM9, RECQL, RAD21L1, MCM8, MMS22L, NSMCE1, AP5S1, POLN, SPIDR*
Ribosomal large subunit biogenesis	5	1.11E-02	*WDR74, EBNA1BP2, AAMP, NLE1, YAE1D1*
Response to ER stress	7	2.11E-02	*HYOU1, CREB3, TMX3, UBA5, PARK2, PDIA4, DDIT3*
Positive regulation of cell growth	7	2.11E-02	*AKT1, NCBP1, HYAL1, FXN, SLC25A33, SFN, CIB1*
Vesicle-mediated transport	10	2.72E-02	*CHMP1A, NAPG, GOLT1B, COPB1, AP3S2, SEC13, SEC22B, ARFGEF1, SAR1A, VPS39*
Translation	27	1.05E-06	*RPL17, RPL19, RPL14, RPL35, RPL38, RPL39, SLC25A21, MRPL10, MRPL13, RPS3A, SLC25A22, RPL12, RPL7A, RSL24D1, SLC25A40, MRPL1, RPS9, RPS8, RPL28, MRPL24, RPS19, RPL13A, UCP2, RPS14, RPS4Y1, RPS13, RPL37A*
Apoptotic process	24	4.52E-04	*DLC1, BCL2L14, HIP1R, LGALS1, HINT2, PIM1, FOXO1, RRAGA, PPP1R13L, VDAC1, RTN3, ZFP36L1, CSNK2A2, CASP6, PDCL3, UBE2D3, DYNLL1, DAD1, IRF1, RIPK2, PAK1, PPP1R15A, CTSH, STPG1*
Regulation of mitophagy	7	5.65E-04	*CSNK2A2, SREBF1, ACTL6A, ZBTB17, RPL28, VDAC1, SREBF2*
Protein folding	15	1.40E-03	*GRPEL2, DNLZ, FKBP1A, LOC783577, PPIF, PFDN2, PDCL3, PFDN1, CCT5, CCT4, PPIA, PFDN5, BAG2, MKKS, LOC100337215*
Protein stabilization	14	2.59E-03	*ATP1B3, CHP1, IFI30, DNLZ, HSPA1A, LAMP1, CCT5, CCT4, PPIB, BAG3, PHB2, TBRG1, WIZ, PIK3R1*
Positive regulation of DNA-directed DNA polymerase activity	4	4.31E-03	*RFC2, CHTF8, CHTF18, DSCC1*
Regulation of protein ubiquitination	8	7.51E-03	*RNF180, BCL10, ADRB2, PELI1, FGFR3, ARRB2, RIPK2, FKBP1A*
Maturation of LSU-rRNA	5	8.27E-03	*EIF6, MRPL1, LAS1L, RPL7A, RPF1*
Cell division	16	1.24E-02	*CLTA, FZR1, KATNB1, DYNLT1, CCNB1, SPC24, FAM83D, CCND3, CCND2, MIS18A, CDC123, BABAM1, SKA2, MAPRE2, WASL, SEPT6*
Response to oxidative stress	9	1.40E-02	*TXNIP, GPX1, MSRA, GCLC, MTF1, NDUFS8, PSIP1, NQO1, PRDX1*
Positive regulation of I-kappaB kinase/NF-kappaB signaling	14	1.60E-02	*BCL10, PELI1, SLC44A2, LITAF, RELA, LGALS1, FKBP1A, TRIM62, PLK2, ZDHHC13, RIPK2, RBCK1, CCDC22, ABL1*
Actin cytoskeleton organization	10	2.23E-02	*CORO1B, FMNL3, PDLIM7, MOB2, RAC3, FLII, TMSB10, PAK1, MKL1, CAPZB*
Cell cycle arrest	8	2.65E-02	*NOTCH2, BRINP1, IRF6, CDC123, TBRG1, IRF1, GAS2L1, THBS1*
Positive regulation of protein targeting to mitochondrion	8	3.99E-02	*CSNK2A2, MICALL2, UBE2D3, MIS18A, ACTL6A, ZBTB17, RPL28, SREBF2*
Embryonic placenta development	4	4.09E-02	*GATA2, HIF1A, EPAS1, CITED2*

GO enrichment analysis of shNT-high zMADs generated several protein transport-related terms, including “endoplasmic reticulum (ER) to Golgi vesicle-mediated transport” and “intracellular protein transport” (Figure 4D, Table 2). shNTs had a 2.7-fold higher expression level for genes that belonged to these categories than the IVFs, whereas the scNTs showed no difference (Figure 4E). The shNT-specific increase in expression of genes with the GO keywords “ER,” “Golgi,” and “vesicle transport” implicates the ER stress response (Hetz et al., 2015). In reality, the GO result from shNT-high zMADs showed the term “response to ER stress” (*p* = 0.02) and the corresponding genes had 3.5-fold higher expression compared to those in IVFs (Figure 4F). In line with this, *ATF6*, which encodes an ER transmembrane glycoprotein that acts as a transcription activator and initiates the unfolded protein response (UPR) during ER stress (Wang et al., 2000), had ~4-fold increased expression in shNTs compared to that in IVFs (*p* = 2.23 × 10^−6^). However, the expression level of *ATF6B*, which functions to reduce expression of ER stress proteins, was not different (*p* = 0.276) between shNTs and IVFs (Thuerauf et al., 2004). KEGG pathway analysis also showed that zMADs were associated with “protein process in ER” related pathways such as “protein processing in endoplasmic reticulum” and “ribosome” in shNT (Supplementary File 2). Therefore, these results suggest that shNTs suffer from ER stress.

### Expression features of epi-driver genes in the shNTs

**Figure 5:**
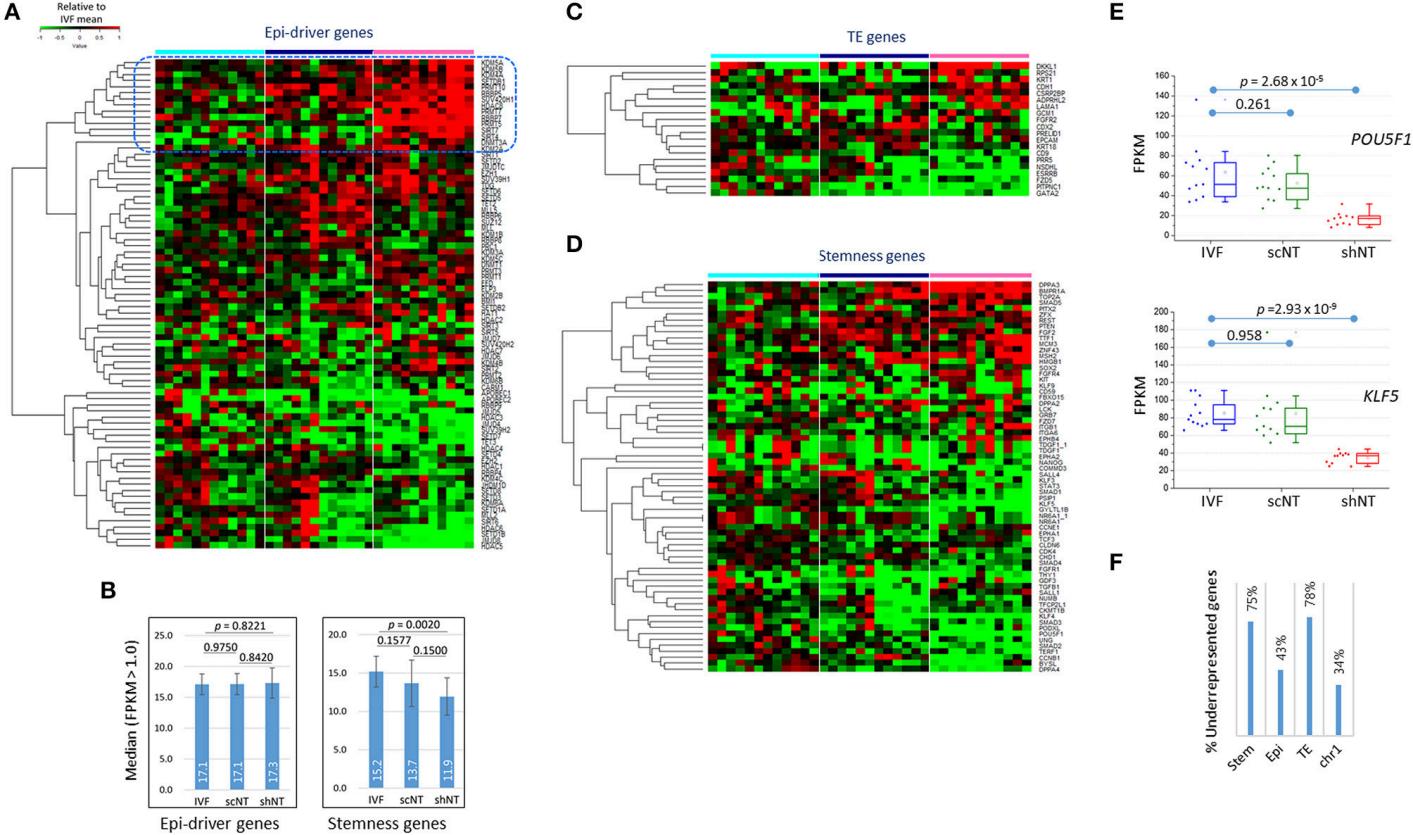
Features of the expression of developmentally important genes in IVF, scNT, and shNT blastocysts. **(A)** Heatmap of relative expression levels against IVF mean of epi-driver genes in IVF (cyan), scNT (blue), and shNT (pink) blastocyst samples. Dotted blue square marks the shNT-high gene cluster where genes implicated in heterochromatin formation and maintenance are concentrated. **(B)** Median expression levels of epi-driver and stemness genes. Error bars, standard deviation. Statistical significances were calculated by paired *t*-test. **(C,D)**. Gene expression patterns of trophectoderm (TE) development **(C)** and stemness **(D)** related genes. **(E)** Expression levels of *POU5F1* and *KLF5* stemness genes in individual blastocyst samples. Statistics, Student's *t*-test. **(F)** Fraction of underrepresented genes among the DEGs (*p* < 0.05) in shNT blastocysts compared with IVF blastocysts.

Of particular interest was whether the expression of those genes important for early development were affected in shNTs. We examined the expression of epi-driver genes, stemness genes, and genes associated with trophectoderm (TE) development (Min et al., 2015; Park et al., 2017). Figure 5A shows the heat-map of expression levels of epi-driver genes in bovine blastocysts. The median expression levels in the three blastocyst groups were very similar (~17) in fragments per kilobase of exon per million mapped fragments (FPKM), showing no significant difference between the groups (*p* > 0.822, paired sample *t*-test; Figure 5B). When we looked closely at the heat-map, the upper part containing genes overrepresented in shNTs was occupied by genes implicated in heterochromatin establishment and maintenance (*KDM5A, KDM5B, SETDB1, SUV420H1, HDAC8, DNMT3A*, and *KDM2A*). This suggests that shNTs treat heterochromatin favorably. In addition, several protein arginine methyltransferase genes (PRMTs; *PRMT9/10, PRMT5*, and *PRMT7*) and sirtuin genes (*SIRT4* and *SIRT7*) were found in that section of the heat-map. Interestingly, of the seven PRMTs that were expressed in cow blastocysts, six were overrepresented in shNTs (Figure S3), although the implication and consequence of this finding to the genome are unknown. In fact, those epi-driver proteins have diverse functions in chromatin modification as writers, erasers, and keepers of the epigenome. Different combinations of the type and amount of epi-drivers can affect a variety of synergic or opposing changes in the configuration and structure of chromatin, reshaping chromatin by either packing or relaxing it. Hence, we could not conjecture to which direction the altered expression of epi-driver genes would lead; we simply know that the altered genes expression of epi-driver indicates altered chromatin compaction or relaxation in shNTs.

### The stemness- and trophectoderm-related genes were underrepresented in the shNTs

Some TE genes, which are important for implantation and placental development and are frequently abnormal in NT embryos (Kang et al., 2002; Koo et al., 2002), were overrepresented (*DKKL1* and *CDH1*; *p* < 0.01) or underrepresented (*ESRRB, GATA2, PITPNC1*, and *CD9*) in shNTs compared to that in IVFs (Figure 5C). In agreement, the GO enrichment analysis using shNT-low zMADs yielded the “embryonic placenta development” term (*GATA2, HIF1A, EPAS1, CITED2*; Table 2). In the IVF-scNT comparison, fewer genes (*GATA2, PITPNC1, EPCAM*, and *CDX2*) were detected even at a lower stringency (*p* < 0.05). Thus, *GATA2* and *PITPNC1* were shared as DEGs in common between shNTs and scNTs. Of the four scNT DEGs, the latter three coincided with those previously identified as TE-related DEGs in scNT blastocysts (Min et al., 2015).

The majority of stemness genes were underrepresented in the shNTs compared to that in the IVFs (Figure 5D). The median expression level of stemness genes was significantly different (*p* = 0.0020) between IVFs and shNTs, but not in other comparisons (Figure 5B). For example, *OCT4* (or *POU5F1*) and *KLF5* expression levels were significantly reduced in shNTs but not in scNTs (Figure 5E). Figure S4 shows the coverage plots for representative stemness genes. In the same context, among the stemness genes that were differently expressed in the shNTs (*p* < 0.05) compared to that in the IVFs, about 75% (24/32) were underrepresented, while only 43% (16/37) of epi-driver genes and 34% (60/178) of chromosome 1 (chr1) genes were underrepresented (Figure 5F). TE genes showed a pattern similar to that shown by the stemness genes, with 78% (7/9) of TE DEGs (*p* < 0.05) underrepresented in shNTs. Because of the small number of DEGs, a comparison of gene expression between scNTs and shNTs was not possible. Meanwhile, there were some stemness genes, such as *BMPR1A* (*p* = 2.43 × 10^−8^) and *DPPA3* (*p* = 1.61 × 10^−4^), that were significantly overrepresented in shNTs (Figure S4).

We additionally performed WGCNA to check the blastocyst-type-specific co-expression networks (modules) and summarized in Figure S5 and Supplementary File 3.

## Discussion

In this study, we analyzed global gene expression pattern in bovine blastocysts generated by various methods, i.e., IVF, scNT, and shNT techniques and compared their transcriptomes to identify the factors induced by manipulation of oocytes or zygotes. The results from PCA and Pearson correlation analysis showed that the shNT vs. IVF correlation was weaker than the scNT vs. IVF correlation (Figure 1E). In line with this, DEGs (*n* = 1873) from the IVF-shNT comparison exceeded the number of DEGs (*n* = 439) from the IVF-scNT comparison (Figure S2). This indicates that the transcriptomic difference between shNTs and IVFs is greater than the difference between scNTs and IVFs. The basic difference between shNTs and scNTs is that the recipients are at different stages of either the cell cycle, i.e., before (metaphase, scNTs) or after fertilization (non-metaphase, shNTs). Because procedures such as machine-aided manipulation and *in vitro* culture following chemical activation were well-controlled, they were not likely to be the principal cause of the difference between shNTs and scNTs. Fertilization brings in a variety of cellular biochemical and biological changes, making it easy to distinguish between oocyte and zygote cytoplasms. Thus, the NT shock may have different effects in the oocyte and the zygote, which manifests as differences in the transcriptomes of shNTs and scNTs (Figure 1).

Since the shNTs had experienced the treatment of Ca^2+^ ionophore and 6-DMAP at the zygote stage, there is a possibility that this post-fertilization stimulation might have given a serious impact on the subsequent development of the zygotes. Alternatively, it could have only moderately affected the zygote or the zygotes might be immune to this second stimulus and thus not much damaged as it was thought. We searched for studies on the effect of repeated activation on the development of mammalian zygotes but failed to find a single one. So, we designed our own experiment to see whether the mouse zygotes, which experienced the repeated chemical activation just like the bovine zygotes, could develop after transfer to pseudo-pregnant mice. The result in the supplementary Figure S1C demonstrated that they could normally develop when observed at 13.5 dpc, which strongly suggests that the second activation is tolerable and not so catastrophic to the bovine zygotes.

The synchronization of the cell cycle has been an important issue for successful cloning in mice, and the use of a zygote recipient does not lead to blastocyst development (McGrath and Solter, 1984a; Wakayama et al., 2000) without such synchronization (Kang et al., 2014). Although cell cycle synchronization was not a consideration in our shNTs, there is still a possibility that the use of a metaphase-synchronized zygote as a recipient may allow the resulting shNTs' transcriptomes to resemble the IVFs'.

Given the weaker correlation between shNTs and IVFs than between scNTs and IVFs, the nuclear transfer-induced physical damage appears to be greater to the zygote than to the oocyte. It would be interesting to know which effect is more devastating on the developing zygote, the perturbation of the cytoskeletal structure or the reduction in cytoplasmic protein/RNA stocks, since fertilization would result in a fundamental change in these two cytoplasmic factors. In reality, among the GO terms generated with the shNT-low zMADs, we found terms directly related to cytoskeletal structure such as “actin cytoskeletal organization” and “microtubule polymerization”, hinting at a disorganization of the cytoskeleton in shNTs.

Another interesting question is whether and by what mechanism the early damage at the zygote could trigger downregulation of stemness and TE genes in blastocysts (Figure 5D). It may be difficult to prove their causal relationship, but we found them to occur with a certain specificity. The genes downregulated in shNTs were limited to stemness and TE gene categories and did not include epi-driver or chr1 genes (Figure 5E). Similarly, considering the nearly equal presence of shNT-high (51%) and shNT-low zMADs (49%) among the 1743 zMADs (Figure 3B), the fact that 75% of stemness- and TE-associated DEGs were underrepresented in shNTs indicates significant bias. Such a seemingly “selective” suppression of developmentally important genes in shNTs may be explained by two possibilities. Firstly, the genes may be expressed during early cleavages in shNT embryos, but then the levels of their transcripts decrease by an unknown manipulation-associated mechanism. Alternatively, the activation of early developmental genes may be delayed in the shNT embryos, possibly due to an unfavorable cellular environment introduced by zygote manipulation. These developmental genes may be programmed to be pre-processed from the zygotic stage for later expression or they may need a balanced embryonic environment (for example, a balanced ratio of the nucleus and cytoplasm) for timely expression. However, manipulation may disturb this design set by the embryo for these genes, resulting in their failed expression. The fertilized ovum is frequently used in genetic studies for pronuclear exchange or substitution via manipulation (McGrath Solter and Solter, 1984b; Craven et al., 2010). Considering both the weak transcriptomic correlation of shNTs with IVFs and the downregulation of developmentally important genes in shNTs, the fertilized ovum is more sensitive than previously thought and is definitely not a stage that could be used in assisted reproductive technology.

Considering the half IVF (the use of the zygote with intact genome) and half scNT (the experience of almost standard NT procedure) characteristics, we had initially thought that the nature of shNTs and their transcriptomes would be midway between those of IVFs and scNTs. This speculation was proven incorrect. shNTs did not resemble IVFs or scNTs as much as we had expected and appeared as a unique entity. Looking at the results, there are also other aspects to be considered. The manipulation experience of shNTs was unlike IVFs, and they did not undergo nuclear reprogramming of the somatic cell genome as did the scNTs. Nevertheless, the inclusion of shNTs in the otherwise simple comparison of transcriptomes of IVFs and scNTs enabled the identification of valuable DEG resources, such as MADs, DADs, and zMADs. Closer inspection of these defined DEGs and related GO terms will significantly improve our understanding of the cellular and molecular events occurring in scNT embryos and our ability to control genome reprogramming for efficient cloning. Lastly, the irrelevance of zygotes as an NT recipient in cloning has been suggested experimentally (McGrath and Solter, 1984a; Wakayama et al., 2000; Kang et al., 2014). The transcriptome analysis we present here provides the rationale for this finding.

## Author contributions

BM: performed all the molecular works and data analysis, and wrote the paper; JP: performed all the embryo manipulations including SCNT and sham NT; Y-KK: designed and supervised the project, and wrote the paper.

### Conflict of interest statement

The authors declare that the research was conducted in the absence of any commercial or financial relationships that could be construed as a potential conflict of interest.

## References

[B1] Akagi S. Matsukawa K. Takahashi S. (2014). Factors affecting the development of somatic cell nuclear transfer embryos in cattle. J. Reprod. Dev. 60, 329–335. 10.1262/jrd.2014-057 25341701 PMC4219988

[B2] Amano T. Tani T. Kato Y. Tsunoda Y. (2001). Mouse cloned from embryonic stem (ES) cells synchronized in metaphase with nocodazole. J. Exp. Zool. 289, 139–145. 10.1002/1097-010X(20010201)289:2<139::AID-JEZ7>3.0.CO;2-6 11169501

[B3] Bourc'his D. Le Bourhis D. Patin D. Niveleau A. Comizzoli P. Renard J.. (2001). Delayed and incomplete reprogramming of chromosome methylation patterns in bovine cloned embryos. Curr. Biol. 11, 1542–1546. 10.1016/S0960-9822(01)00480-8 11591324

[B4] Bowles E. J. Tecirlioglu R. T. French A. J. Holland M. K. St John J. C. (2008). Mitochondrial DNA transmission and transcription after somatic cell fusion to one or more cytoplasts. Stem Cells 26, 775–782. 10.1634/stemcells.2007-0747 18192233

[B5] Craven L. Tuppen H. A. Greggains G. D. Harbottle S. J. Murphy J. L. Cree L. M.. (2010). Pronuclear transfer in human embryos to prevent transmission of mitochondrial DNA disease. Nature 465, 82–85. 10.1038/nature08958 20393463 PMC2875160

[B6] Dumollard R. Ward Z. Carroll J. Duchen M. R. (2007). Regulation of redox metabolism in the mouse oocyte and embryo. Development 134, 455–465. 10.1242/dev.02744 17185319

[B7] Eggan K. Akutsu H. Loring J. Jackson-Grusby L. Klemm M. Rideout W. M.. (2001). Hybrid vigor, fetal overgrowth, and viability of mice derived by nuclear cloning and tetraploid embryo complementation. Proc. Natl. Acad. Sci. U.S.A. 98, 6209–6214. 10.1073/pnas.101118898 11331774 PMC33447

[B8] Hetz C. Chevet E. Oakes S. A. (2015). Proteostasis control by the unfolded protein response. Nat. Cell. Biol. 17, 829–838. 10.1038/ncb3184 26123108 PMC5546321

[B9] Hill J. R. Winger Q. A. Long C. R. Looney C. R. Thompson J. A. Westhusin M. E. (2000). Development rates of male bovine nuclear transfer embryos derived from adult and fetal cells [In Process Citation]. Biol. Reprod. 62, 1135–1140. 10.1095/biolreprod62.5.1135 10775159

[B10] Hogan B. (1994). Manipulating the Mouse Embryo: A Laboratory Manual. Plainview, NY: Cold Spring Harbor Laboratory Press.

[B11] Howlett S. K. Barton S. C. Surani M. A. (1987). Nuclear cytoplasmic interactions following nuclear transplantation in mouse embryos. Development 101, 915–923. 3503704 10.1242/dev.101.4.915

[B12] Hua S. Zhang H. Su J. M. Zhang T. Quan F. S. Liu J.. (2011). Effects of the removal of cytoplasm on the development of early cloned bovine embryos. Anim. Reprod. Sci. 126, 37–44. 10.1016/j.anireprosci.2011.05.002 21632190

[B13] Huang da W. Sherman B. T. Lempicki R. A. (2009). Systematic and integrative analysis of large gene lists using DAVID bioinformatics resources. Nat. Protoc. 4, 44–57. 10.1038/nprot.2008.211 19131956

[B14] Humpherys D. Eggan K. Akutsu H. Hochedlinger K. Rideout W. M. III. Biniszkiewicz D.. (2001). Epigenetic instability in ES cells and cloned mice. Science 293, 95–97. 10.1126/science.1061402 11441181

[B15] Kang E. Wu G. Ma H. Li Y. Tippner-Hedges R. Tachibana M.. (2014). Nuclear reprogramming by interphase cytoplasm of two-cell mouse embryos. Nature 509, 101–104. 10.1038/nature13134 24670652 PMC4124901

[B16] Kang Y. K. Koo D. B. Park J. S. Choi Y. H. Chung A. S. Lee K. K.. (2001). Aberrant methylation of donor genome in cloned bovine embryos. Nat. Genet. 28, 173–177. 10.1038/88903 11381267

[B17] Kang Y. K. Lee K. K. Han Y. M. (2003). Reprogramming DNA methylation in the preimplantation stage: peeping with Dolly's eyes. Curr. Opin. Cell. Biol. 15, 290–295. 10.1016/S0955-0674(03)00031-0 12787770

[B18] Kang Y. K. Park J. S. Koo D. B. Choi Y. H. Kim S. U. Lee K. K.. (2002). Limited demethylation leaves mosaic-type methylation states in cloned bovine pre-implantation embryos. Embo. J. 21, 1092–1100. 10.1093/emboj/21.5.1092 11867537 PMC125883

[B19] Kim D. Pertea G. Trapnell C. Pimentel H. Kelley R. Salzberg S. L. (2013). TopHat2: accurate alignment of transcriptomes in the presence of insertions, deletions and gene fusions. Genome Biol. 14:R36. 10.1186/gb-2013-14-4-r36 23618408 PMC4053844

[B20] Koo D. B. Kang Y. K. Choi Y. H. Park J. S. Kim H. N. Oh K. B.. (2002). Aberrant allocations of inner cell mass and trophectoderm cells in bovine nuclear transfer blastocysts. Biol. Reprod. 67, 487–492. 10.1095/biolreprod67.2.487 12135886

[B21] Kwon D. Ji M. Lee S. Seo K. W. Kang K. S. (2017). Reprogramming enhancers in somatic cell nuclear transfer, ipsc technology, and direct conversion. Stem Cell Rev. 13, 24–34. 10.1007/s12015-016-9697-x 27817181

[B22] Kwon S. Jeong S. Park J. S. Kang Y. K. (2015). Quantifying difference in gene expression profile between bovine blastocysts derived by *in vitro* fertilization and somatic cell nuclear transfer. Gene Expr. Patterns 19, 14–20. 10.1016/j.gep.2015.05.005 26101995

[B23] Lagutina I. Lazzari G. Duchi R. Turini P. Tessaro I. Brunetti D.. (2007). Comparative aspects of somatic cell nuclear transfer with conventional and zona-free method in cattle, horse, pig and sheep. Theriogenology 67, 90–98. 10.1016/j.theriogenology.2006.09.011 17081599

[B24] Langfelder P. Horvath S. (2008). WGCNA: an R package for weighted correlation network analysis. BMC Bioinformatics 9:559. 10.1186/1471-2105-9-559 19114008 PMC2631488

[B25] Lanza R. P. Cibelli J. B. Blackwell C. Cristofalo V. J. Francis M. K. Baerlocher G. M.. (2000). Extension of cell life-span and telomere length in animals cloned from senescent somatic cells. Science 288, 665–669. 10.1126/science.288.5466.665 10784448

[B26] Lemasters J. J. (2005). Selective mitochondrial autophagy, or mitophagy, as a targeted defense against oxidative stress, mitochondrial dysfunction, and aging. Rejuvenat. Res. 8, 3–5. 10.1089/rej.2005.8.3 15798367

[B27] McGrath J. Solter D. (1984a). Inability of mouse blastomere nuclei transferred to enucleated zygotes to support development *in vitro*. Science 226, 1317–1319. 10.1126/science.6542249 6542249

[B28] McGrath J. Solter D. (1984b). Completion of mouse embryogenesis requires both the maternal and paternal genomes. Cell 37, 179–183. 10.1016/0092-8674(84)90313-1 6722870

[B29] Min B. Cho S. Park J. S. Jeon K. Kang Y. K. (2016). The HIST1 locus escapes reprogramming in cloned bovine embryos. G3 6, 1365–1371. 10.1534/g3.115.026666 26976441 PMC4856087

[B30] Min B. Cho S. Park J. S. Lee Y. G. Kim N. Kang Y. K. (2015). Transcriptomic features of bovine blastocysts derived by somatic cell nuclear transfer. G3 5, 2527–2538. 10.1534/g3.115.020016 26342001 PMC4683625

[B31] Min B. Park J. S. Jeon K. Kang Y. K. (2017). Characterization of X-chromosome gene expression in bovine blastocysts derived by *in vitro* fertilization and somatic cell nuclear transfer. Front. Genet. 8:42. 10.3389/fgene.2017.00042 28443134 PMC5385346

[B32] Niemann H. Lucas-Hahn A. (2012). Somatic cell nuclear transfer cloning: practical applications and current legislation. Reprod. Domest. Anim. 47, 2–10. 10.1111/j.1439-0531.2012.02121.x 22913555

[B33] Niemann H. Tian X. C. King W. A. Lee R. S. (2008). Epigenetic reprogramming in embryonic and foetal development upon somatic cell nuclear transfer cloning. Reproduction 135, 151–163. 10.1530/REP-07-0397 18239046

[B34] Ogura A. Inoue K. Wakayama T. (2013). Recent advancements in cloning by somatic cell nuclear transfer. Philos. Trans. R Soc. Lond. B Biol. Sci. 368:20110329 10.1098/rstb.2011.0329 23166393 PMC3539358

[B35] Ono Y. Shimozawa N. Ito M. Kono T. (2001). Cloned mice from fetal fibroblast cells arrested at metaphase by a serial nuclear transfer. Biol. Reprod. 64, 44–50. 10.1095/biolreprod64.1.44 11133657

[B36] Panda S. K. George A. Saha A. P. Sharma R. Manik R. S. Chauhan M. S.. (2011). Effect of cytoplasmic volume on developmental competence of buffalo (*Bubalus bubalis*) embryos produced through hand-made cloning. Cell. Reprogram. 13, 257–262. 10.1089/cell.2010.0096 21563942

[B37] Park J. S. Jeong Y. S. Shin S. T. Lee K. K. Kang Y. K. (2007). Dynamic DNA methylation reprogramming: active demethylation and immediate remethylation in the male pronucleus of bovine zygotes. Dev. Dyn. 236, 2523–2533. 10.1002/dvdy.21278 17676637

[B38] Park M. Min B. Jeon K. Cho S. Park J. S. Kim J.. (2017). Age-associated chromatin relaxation is enhanced in Huntington's disease mice. Aging 9, 803–822. 10.18632/aging.101193 28288000 PMC5391233

[B39] Reik W. Dean W. Walter J. (2001). Epigenetic reprogramming in mammalian development. Science 293, 1089–1093. 10.1126/science.1063443 11498579

[B40] Smith Z. D. Sindhu C. Meissner A. (2016). Molecular features of cellular reprogramming and development. Nat. Rev. Mol. Cell. Biol. 17, 139–154. 10.1038/nrm.2016.6 26883001

[B41] Surani M. A. (2001). Reprogramming of genome function through epigenetic inheritance. Nature 414, 122–128. 10.1038/35102186 11689958

[B42] Thuerauf D. J. Morrison L. Glembotski C. C. (2004). Opposing roles for ATF6α and ATF6β in endoplasmic reticulum stress response gene induction. J. Biol. Chem. 279, 21078–21084. 10.1074/jbc.M400713200 14973138

[B43] Trapnell C. Roberts A. Goff L. Pertea G. Kim D. Kelley D. R.. (2012). Differential gene and transcript expression analysis of RNA-seq experiments with TopHat and Cufflinks. Nat. Protoc. 7, 562–578. 10.1038/nprot.2012.016 22383036 PMC3334321

[B44] Wakayama T. Tateno H. Mombaerts P. Yanagimachi R. (2000). Nuclear transfer into mouse zygotes. Nat. Genet. 24, 108–109. 10.1038/72749 10655051

[B45] Wang Y. Shen J. Arenzana N. Tirasophon W. Kaufman R. J. Prywes R. (2000). Activation of ATF6 and an ATF6 DNA binding site by the endoplasmic reticulum stress response. J. Biol. Chem. 275, 27013–27020. 10.1074/jbc.M003322200 10856300

[B46] Xue Z. Huang K. Cai C. Cai L. Jiang C. Y. Feng Y.. (2013). Genetic programs in human and mouse early embryos revealed by single-cell RNA sequencing. Nature 500, 593–597. 10.1038/nature12364 23892778 PMC4950944

[B47] Yeo S. Lee K. K. Han Y. M. Kang Y. K. (2005). Methylation changes of lysine 9 of histone H3 during preimplantation mouse development. Mol. Cells 20, 423–428. 16404159

