# Supplementary material for: Determination of Oocyte-Manipulation, Zygote-Manipulation, and Genome-Reprogramming Effects on the Transcriptomes of Bovine Blastocysts
Source: Front Genet. 2018 Apr 24;9:143. doi: 10.3389/fgene.2018.00143 (PMC5928200; doi:10.3389/fgene.2018.00143)
Supplement: Supplementary file 4 [file Image_1.pdf]

Supplementary Figures

A

	IVC	Cleaved to 2-, 4-, 8-cell (%)	Blastocysts from 8-cell (%)
IVF	512	26, 98, 251 (73.2)	133/251 (53.0)
scNT	309	15, 39, 66 (38.8)	44/66 (66.7)
shNT	497	95, 210, 130 (87.5)	40/130 (30.8)

B

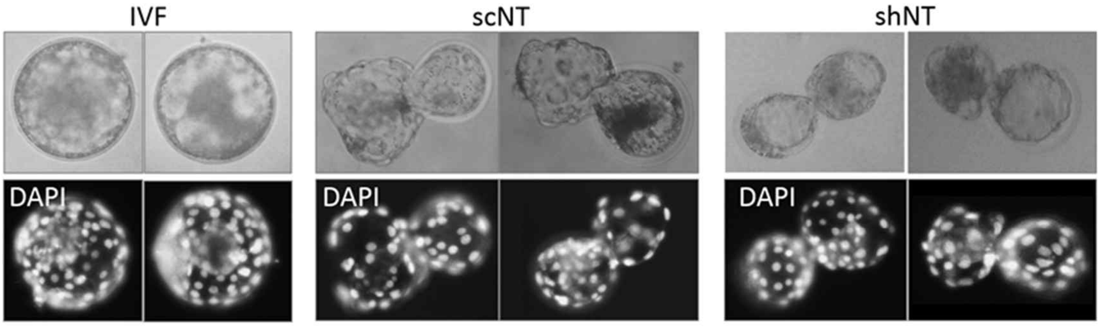

C

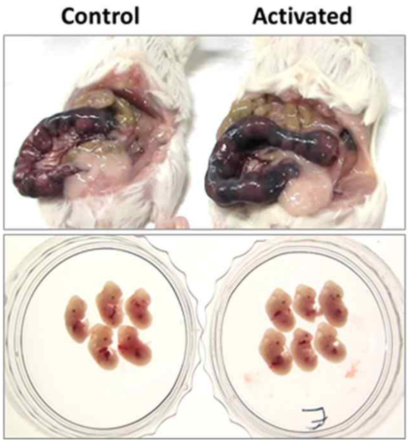

	No. of 1-cell embryos	No. of 2-cell embryos transferred	No. of Fetus (%)
Control	15	15	5 (33.3)
Activated	17	16	6 (37.5)

Supplementary Figure S1.
A. In vitro developmental rates of the IVF, scNT, and shNT groups.
B. Quality assessment of blastocysts generated by IVF, scNT or shNT using live Hoechst staining.
C. Mouse fetuses generated by repeated embryo activation procedure and normally fertilized embryos. Number of embryos collected and transferred to surrogate mothers and number of fetus normally developed are summarized on the right.

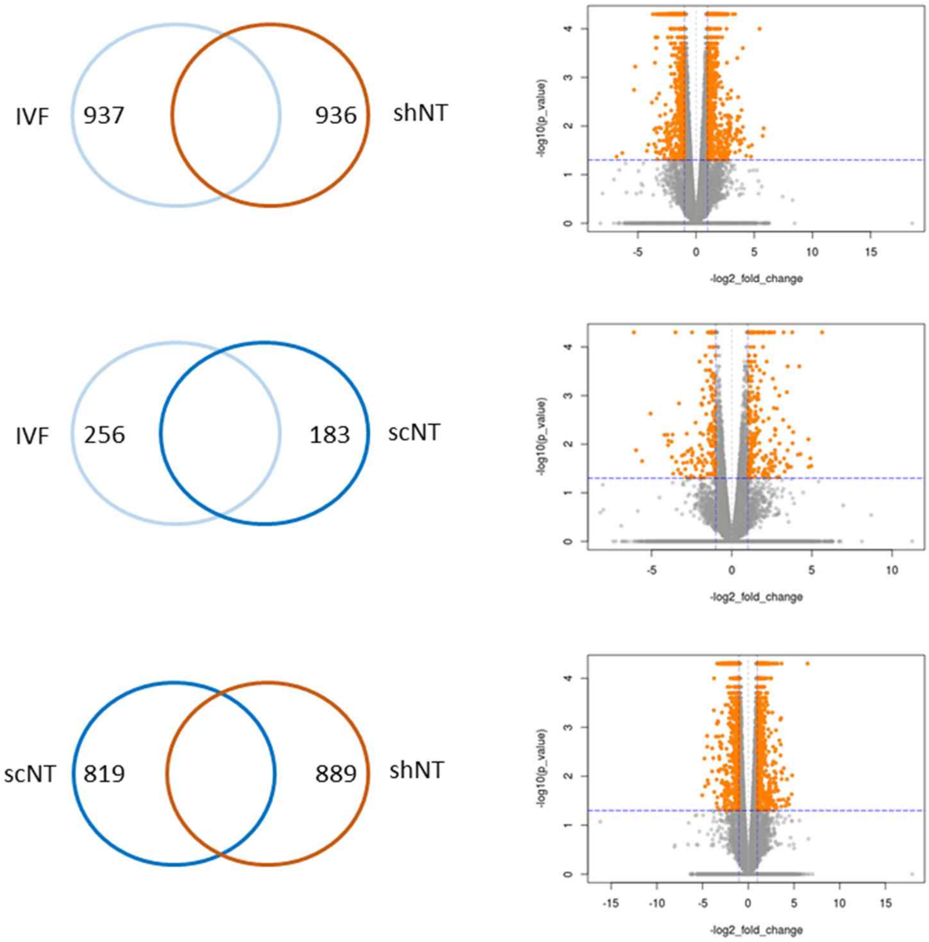

Supplementary Figure S2. Identification of differentially expressed genes (DEGs) between blastocyst groups. Left, Venn diagrams showing the number of DEGs (fold-change > 2, *p*-value < 0.05) between the denoted blastocyst groups. Right, corresponding volcano plots. DEGs are colored orange.

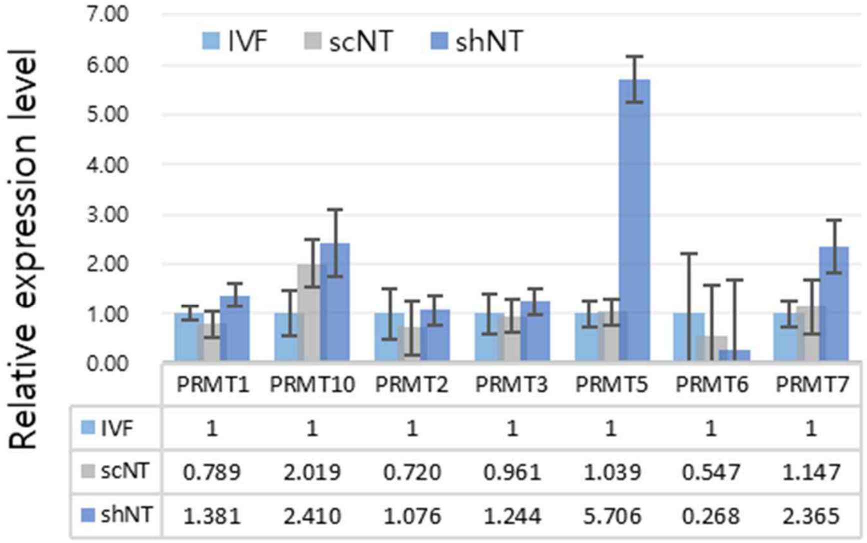

Supplementary Figure S3. Relative expression levels of protein arginine methyltransferases (PRMTs) in IVF, scNT, and shNT blastocysts. Of seven PRMTs expressed in blastocysts, six were overrepresented in shNT blastocysts. Relative expression levels against IVFs are denoted below. Error bars, standard deviation. Statistics, paired *t*-test.

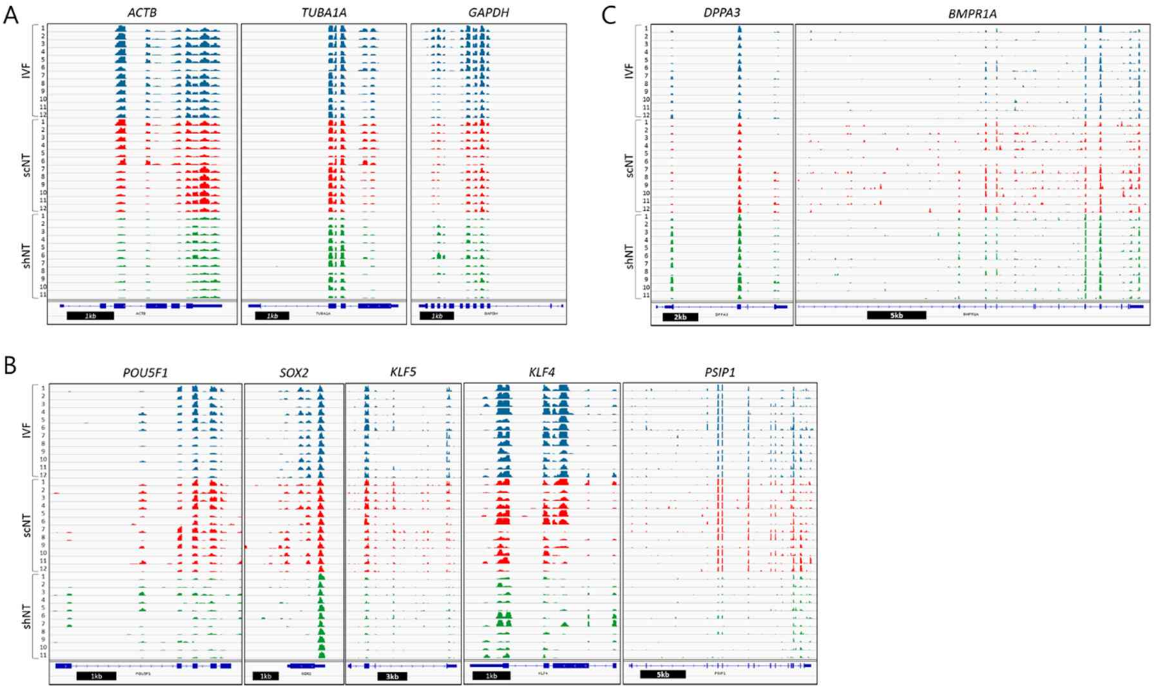

Supplementary Figure S4. Visualization of read coverages of housekeeping genes (A) and stemness genes (B and C) expressed in IVF, scNT, and shNT blastocysts on IGV.

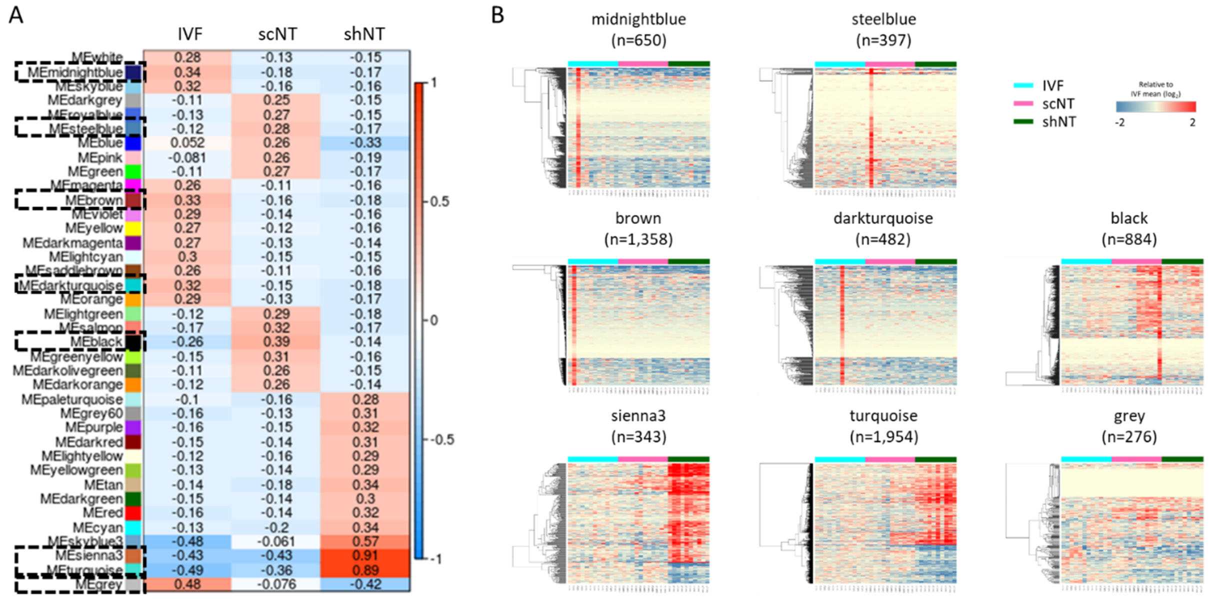

Supplementary Figure S5. Weighted gene co-expression network analysis (WGCNA). (A) Correlation between co-expression gene modules and blastocyst groups. Selected modules for B were marked with black dotted boxes. A scale bar on the right indicates the correlation values between modules and blastocyst types; Orange = positive correlation, Blue = negative correlation, White = no correlation. (B) Heatmaps of member gene expression patterns in selected modules (black boxed modules in A).
